# Soil parameters, land use, and geographical distance drive soil bacterial communities along a European transect

**DOI:** 10.1038/s41598-018-36867-2

**Published:** 2019-01-24

**Authors:** Pierre Plassart, Nicolas Chemidlin Prévost-Bouré, Stéphane Uroz, Samuel Dequiedt, Dorothy Stone, Rachel Creamer, Robert I. Griffiths, Mark J. Bailey, Lionel Ranjard, Philippe Lemanceau

**Affiliations:** 1 Agroécologie, AgroSup Dijon, INRA, Univ. Bourgogne, Univ. Bourgogne Franche-Comté, F-21000 Dijon, France; 2 UMR 1136 Interactions Arbres Micro-organismes, INRA Univ Lorraine, F-54280 Champenoux, France; 3 TEAGASC, Johnstown Castle, Wexford, Ireland; 4 Present Address: Wageningen University and Research, Wageningen, The Netherlands; 5 Centre for Ecology & Hydrology, Benson Lane, Crowmarsh Gifford, Wallingford, UK

## Abstract

To better understand the relationship between soil bacterial communities, soil physicochemical properties, land use and geographical distance, we considered for the first time ever a European transect running from Sweden down to Portugal and from France to Slovenia. We investigated 71 sites based on their range of variation in soil properties (pH, texture and organic matter), climatic conditions (Atlantic, alpine, boreal, continental, Mediterranean) and land uses (arable, forest and grassland). 16S rRNA gene amplicon pyrosequencing revealed that bacterial communities highly varied in diversity, richness, and structure according to environmental factors. At the European scale, taxa area relationship (TAR) was significant, supporting spatial structuration of bacterial communities. Spatial variations in community diversity and structure were mainly driven by soil physicochemical parameters. Within soil clusters (k-means approach) corresponding to similar edaphic and climatic properties, but to multiple land uses, land use was a major driver of the bacterial communities. Our analyses identified specific indicators of land use (arable, forest, grasslands) or soil conditions (pH, organic C, texture). These findings provide unprecedented information on soil bacterial communities at the European scale and on the drivers involved; possible applications for sustainable soil management are discussed.

## Introduction

Monitoring soil microbial communities represents a major challenge to better valorize soil resources and to implement a more sustainable management of soils in agriculture and forestry^1,2^. A more thorough knowledge of the geographical distribution of microbial communities across different soil types and the identification of potential biotic indicators of changes are critical to better understand the ways soils and land uses impact microbial diversity under different climates. These indicators will help managers devise strategies for monitoring microbial diversity and soil functioning for end users and policy makers^3^.

A large debate on the relative importance of soil properties and plants on belowground biodiversity has been running over the last decades^4–6^. Local- to continental-scale studies have suggested that soil properties (e.g. the pH) are major drivers of the structure and diversity of soil microbial communities, with plant communities and land use as secondary confounded correlates^7–15^. However, more locally focussed experiments revealed that in certain soils, microbial communities are also strongly affected by plants species, developmental stage and phenology^16–18^. These observations are consistent with the recent proposal by several authors that a core microbiome associated with plant roots is specific to the host genotype and includes populations beneficial for plant growth and health^19–22^. However, the rhizosphere effect is known to vary according to the soil type, even for a given plant species^16,23,24^. Plants indeed recruit their rhizosphere microbiota from the soil reservoir^25^, whose biodiversity varies among soils as indicated by biogeographical studies^12^. Moreover, as a given land use can be found in various types of soils, it is essential for sustainable soil management to better understand the effects of land use on microbial communities specific to the soil type. Furthermore, specific indicators of land use *vs*. soil type are needed. So far, most of the biogeographic surveys have focused on broad-scale patterns and drivers, and few attempts have been made to disentangle the effects of different land use on similar soils, and *vice versa*. A key issue with broad landscape surveys is that human land management is strongly determined by the prevailing soil and climatic properties of the environment. With the onset of agricultural development, human populations have shaped their environment locally by converting the most fertile and easily manageable soils to agriculture and grassland, leaving infertile and rocky soils to forest, heathland and bog^26,27^. At a small scale, this phenomenon confounds gradients of soil properties with land management, masking the primary drivers of soil communities. At a larger spatial scale, these parallel events have led to the distribution of given land uses on very different soil types, although certain soil types are preferentially used for arable land, grassland or forest. For example, grassland tends to occur across a broader range of soil conditions than arable land. Forests are usually developed on nutrient-poor soils, which vary from very acidic to neutral pH values.

This study addresses these shortcomings by specifically determining the relative effects of land use and soil properties on bacterial diversity and structure in a large number of soil samples collected and characterised across the first European transect for soil microbes. This transect ran from Sweden down to Portugal and from France to Slovenia. The distribution of different land uses on various soil conditions across this transect allowed us to test the respective effects of these two categories of drivers on soil bacterial communities. The sampled sites represented three major land uses (arable, forest, grassland) across a range of soils chosen for their contrasting pH values, textures, and organic matter contents, and covering a broad range of climatic conditions. We analysed these soil samples for their physicochemical properties, and for their bacterial diversity and community composition by 16S rRNA gene amplicon pyrosequencing. We used a k-mean classification analysis to group the soils on the basis of their physicochemical properties and land use into homogeneous clusters, and then test if a potential land use effect was observable in these clusters. Finally, we performed an indicator species analysis to identify the genera indicative of particular soil conditions and/or land uses.

## Results and Discussion

### Soil properties

**Figure 1:**
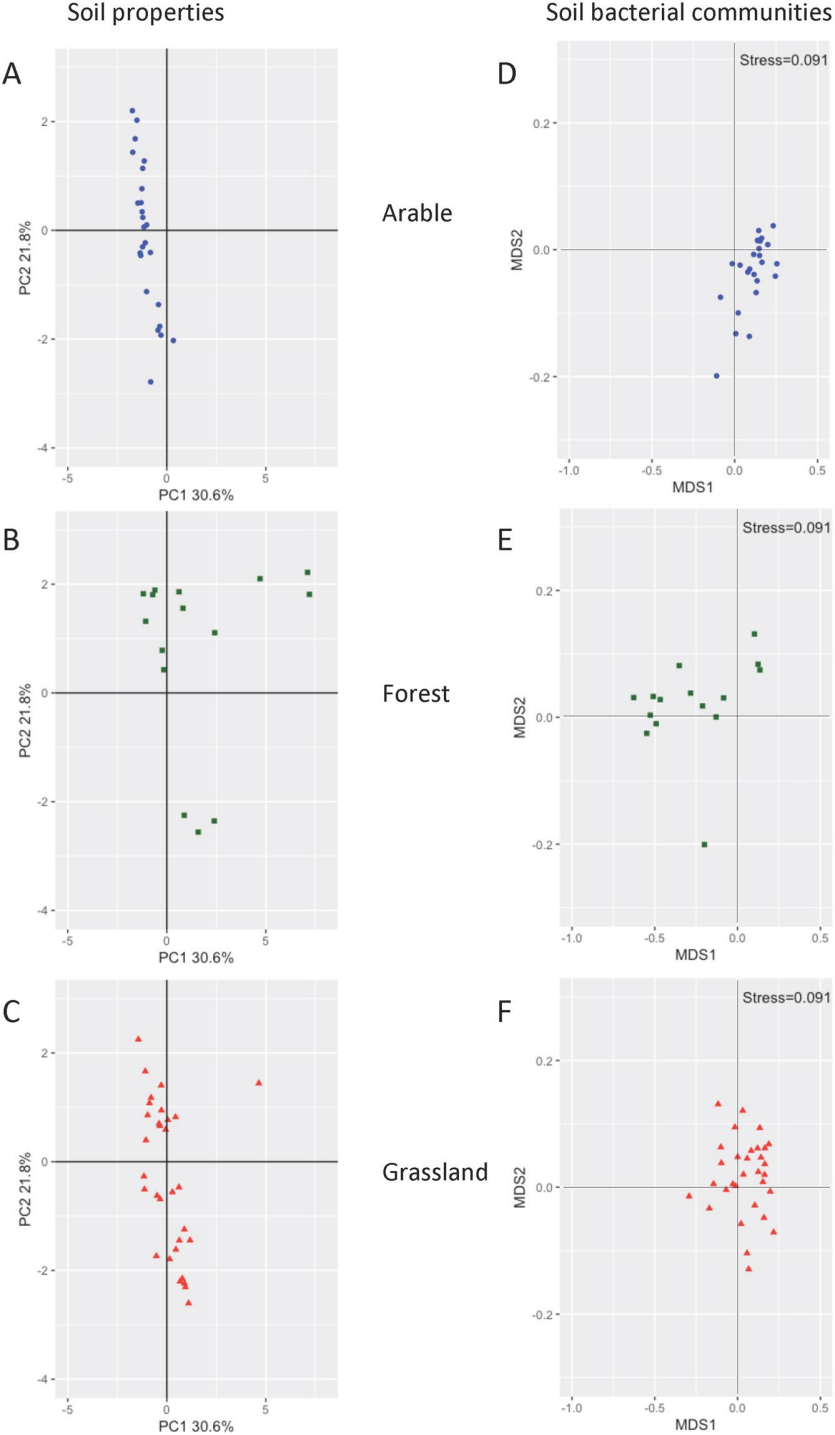
Multivariate analyses of the soil physicochemical properties (**A**–**C**) and bacterial communities (**D**–**F**) for each land use: arable (**A**,**D**), forest (**B**,**E**), grassland (**C**,**F**). The principal component analyses generated from the physicochemical characteristics (**A**–**C**) or relative abundance of all the detected taxa (**D**–**F**) illustrate the diversity of soil properties and bacterial communities according to the three land uses tested. Each symbol represents a sampling site.

According to physicochemical analyses, the 71 soil samples collected across Europe differed strongly in terms of pH, texture, density, and C or N contents (Table 1). pH ranged from 3.7 to 8.2, textures varied from very fine (70% clay in a French arable soil) to coarse (94% sand in a Danish grassland), and organic C content ranged from 0.4% (arable soil, France) to 33% (forest soil, Sweden). The sites were all distinguishable from one another based on soil properties. Studies on the effect of land use or soil properties on bacterial communities had already been performed at a continental scale^7,9,15^, but our study characterises bacterial communities at the European scale for the first time^28^. The sampling design was representative of the variations in soil properties, *i.e*. organic matter content, pH and texture, across different geographical/climatic zones in Europe, and it covered three land uses (arable, forestry, grassland, Table 1). This extensive sampling strategy was consistent in investigating different environmental characteristics regarding the multivariate analyses (Fig. 1A–F). Such a strategy minimises possible biases related to land use. Highly fertile soils are indeed commonly dedicated to agriculture, while nutrient-poor and rocky soils are usually associated to forest as highlighted in previous studies^7,9,12,15^. Thus, our sampling strategy was adequate for disentangling the effects of land use from those of soil properties on bacterial diversity across Europe.

**Table 1:** Sampling sites characteristics.

Sample	pH	pH class	Org C (%)	P (mg/L)	Org C Class	Bulk density	Total C(%)	Total N(%)	FAO texture class	Land Use	Climatic zone	k-mean clusters
DMK_2	4.92	<5	0.99	135.44	<2%	1.149	1.63	0.995	C	Grass	Atlantic	1
DMK_5	6.01	5 to 7	1.08	151.94	<2%	1.456	1.36	1.08	C	Arable	Atlantic	1
FRA_1	5.36	5 to 7	0.59	43.60	<2%	1.489	0.794	0.594	M	Arable	Atlantic	1
FRA_2	5.44	5 to 7	4.18	30.45	2–15%	0.748	4.56	4.18	M	Grass	Atlantic	1
FRA_4	6.29	5 to 7	0.45	109.75	<2%	1.573	0.577	0.45	C	Arable	Atlantic	1
FRA_17	5.41	5 to 7	2.79	129.06	2–15%	0.99	2.4	2.79	C	Forestry	Atlantic	1
FRA_18	5.33	5 to 7	4.4	23.90	2–15%	0.897	5.02	4.4	M	Forestry	Atlantic	1
FRA_19	4.92	<5	4.26	12.32	2–15%	0.799	4.38	4.26	M	Forestry	Atlantic	1
FRA_20	6.69	5 to 7	1.24	92.34	<2%	1.283	1.48	1.24	M	Arable	Atlantic	1
GER_1	6.5	5 to 7	2.89	451.46	2–15%	na	4.2	2.89	C	Arable	Atlantic	1
GER_10	4.63	<5	3.66	15.24	2–15%	0.89	5.06	3.66	C	Forestry	Continental	1
IRE_1	5.81	5 to 7	1.95	56.40	<2%	1.364	2.51	1.95	M	Grass	Atlantic	1
IRE_2	6.59	5 to 7	2.03	48.05	2–15%	1.411	2.35	2.03	M	Grass	Atlantic	1
IRE_3	7.63	>7	1.5	56.67	<2%	1.273	1.84	1.5	M	Arable	Atlantic	1
IRE_4	7.75	>7	1.89	108.81	<2%	1.363	3.73	1.89	M	Arable	Atlantic	1
IRE_5	5.46	5 to 7	3.95	52.08	2–15%	0.935	4.66	3.95	M	Grass	Atlantic	1
IRE_6	7.55	>7	2.06	145.47	2–15%	0.848	2.24	2.06	C	Arable	Atlantic	1
ITA_1	5.2	5 to 7	4.77	26.32	2–15%	0.95	5.6	4.77	F	Grass	Alpine	1
NLD_1	7.8	>7	3.02	166.35	2–15%	1.341	3.71	3.02	F	Arable	Atlantic	1
NLD_2	8.13	>7	1.14	94.73	<2%	1.474	2.4	1.14	M	Arable	Atlantic	1
SWZ_1	6	5 to 7	3.42	314.31	2–15%	1.11	4.03	3.42	M	Grass	Alpine	1
SWZ_4	7.78	>7	3.17	27.33	2–15%	1.028	4.5	3.17	F	Arable	Alpine	1
UKM_2	7.52	>7	3.17	237.58	2–15%	0.983	3.71	3.17	M	Arable	Atlantic	1
UKM_5	4.31	<5	2.73	10.14	2–15%	1.024	3.24	2.73	C	Forestry	Atlantic	1
UKM_6	7.01	>7	3.78	98.26	2–15%	na	5.03	3.78	M	Grass	Atlantic	1
UKM_8	5.31	5 to 7	1.9	61.00	<2%	0.87	3.22	1.9	C	Grass	Atlantic	1
UKM_10	5.58	5 to 7	3.06	31.28	2–15%	0.92	3.95	3.06	C	Grass	Atlantic	1
FRA_3	5.19	5 to 7	5.54	197.31	2–15%	0.714	6.54	5.54	M	Grass	Atlantic	2
FRA_7	5.15	5 to 7	7.41	55.87	2–15%	0.824	9.49	7.41	M	Grass	Continental	2
FRA_8	5.35	5 to 7	5.13	41.03	2–15%	0.909	6.14	5.13	M	Grass	Continental	2
FRA_12	8.05	>7	2.19	22.99	2–15%	1.451	6.61	2.19	F	Arable	Continental	2
FRA_13	6.89	5 to 7	7.61	71.00	2–15%	0.778	8.09	7.61	F	Grass	Continental	2
GER_3	7.47	>7	8.89	70.81	2–15%	na	9.1	8.89	F	Grass	Continental	2
GER_4	7.36	>7	11.3	14.44	2–15%	na	12	11.3	F	Forestry	Continental	2
GER_8	7.42	>7	5.66	177.65	2–15%	na	6.13	5.66	F	Grass	Continental	2
GER_9	3.96	<5	18.6	16.93	>15%	0.301	16.1	18.6	M	Forestry	Continental	2
SLO_1	7.24	>7	9.49	77.23	2–15%	0.719	10.7	9.49	M	Grass	Alpine	2
SLO_2	6	5 to 7	6.17	6.77	2–15%	0.789	6.9	6.17	F	Grass	Alpine	2
SLO_3	7.65	>7	11.3	36.00	2–15%	0.77	12.5	11.3	M	Grass	Alpine	2
SLO_5	7.08	>7	15.9	18.58	>15%	0.425	15.9	15.9	F	Forestry	Alpine	2
SWE_4	3.7	<5	8.21	135.45	2–15%	0.209	9.89	8.21	M	Forestry	Continental	2
SWE_5	4.03	<5	9.46	90.76	2–15%	na	10.6	9.46	M	Forestry	Alpine	2
SWZ_2	5.14	5 to 7	6.56	60.12	2–15%	0.756	7.54	6.56	F	Grass	Alpine	2
SWZ_3	6.54	5 to 7	5.81	101.63	2–15%	0.835	6.78	5.81	F	Grass	Alpine	2
SWZ_5	5.5	5 to 7	5.04	196.62	2–15%	0.899	6.26	5.04	M	Grass	Alpine	2
UKM_1	6.62	5 to 7	7.15	21.57	2–15%	0.82	7.29	7.15	F	Forestry	Atlantic	2
UKM_3	7.42	>7	8.48	22.64	2–15%	0.694	9.7	8.48	F	Grass	Atlantic	2
UKM_7	7.68	>7	5.37	99.35	2–15%	0.729	8.27	5.37	F	Arable	Atlantic	2
UKM_9	7.31	>7	7.8	136.82	2–15%	0.779	8.79	7.8	F	Grass	Atlantic	2
UKM_12	7.52	>7	6.73	26.89	2–15%	0.787	9.05	6.73	F	Grass	Atlantic	2
UKM_13	5.12	5 to 7	5.51	24.26	2–15%	0.684	6.83	5.51	M	Grass	Atlantic	2
DMK_1	4.48	<5	16.5	52.92	>15%	na	23.8	16.5	Org	Forestry	Atlantic	3
FRA_16	5.15	5 to 7	19	36.51	>15%	0.477	20.1	19	Org	Grass	Continental	3
SWE_3	3.97	<5	32.1	77.02	>15%	0.271	31.5	32.1	Org	Forestry	Boreal	3
SWE_7	3.93	<5	31.4	169.09	>15%	na	33	31.4	Org	Forestry	Boreal	3
DMK_3	6.03	5 to 7	1.59	386.06	<2%	1.246	2.02	1.59	C	Arable	Continental	4
DMK_4	7.88	>7	1.01	36.66	<2%	1.336	1.38	1.01	M	Arable	Continental	4
FRA_5	7.84	>7	2.32	24.13	2–15%	1.081	2.72	2.32	VF	Arable	Atlantic	4
FRA_9	5.75	5 to 7	2.14	244.71	2–15%	0.978	2.4	2.14	M	Arable	Continental	4
FRA_10	8.23	>7	1.72	3.64	<2%	1.43	6.06	1.72	MF	Arable	Mediterranean	4
FRA_11	7.55	>7	1.73	48.44	<2%	1.237	2.17	1.73	M	Grass	Mediterranean	4
FRA_14	6.54	5 to 7	2	34.78	2–15%	1.171	2.26	2	F	Arable	Continental	4
FRA_15	7.55	>7	2.33	35.10	2–15%	1.274	2.87	2.33	F	Grass	Continental	4
GER_6	6.95	5 to 7	1.55	126.87	<2%	1.221	1.87	1.55	M	Arable	Continental	4
GER_12	6.37	5 to 7	3.58	17.13	2–15%	na	4.31	3.96	MF	Grass	Continental	4
GER_13	6.79	5 to 7	2.03	96.40	2–15%	1.069	2.23	2.03	M	Arable	Continental	4
ITA_3	6.4	5 to 7	1.24	17.87	<2%	1.328	1.37	1.24	MF	Grass	Continental	4
ITA_4	7.01	>7	1.53	57.22	<2%	1.568	1.84	1.53	M	Arable	Continental	4
ITA_7	7.35	>7	2.29	316.01	2–15%	1.218	2.94	2.29	M	Arable	Continental	4
POR_2	5.16	5 to 7	1.36	69.49	<2%	1.009	1.73	1.36	M	Forestry	Mediterranean	4
POR_4	6.1	5 to 7	0.83	31.45	<2%	1.493	1.19	0.833	M	Arable	Mediterranean	4

The texture classes are Coarse (C), Medium (M), Medium-Fine (MF), Fine (F), Very Fine (VF), and Organic (Org). The following abbreviations have been used: Org C, Organic carbon; Org C Class, Organic C class; P, phosphrous; Total C, total carbon; Total N, total nitrogen. The last column corresponds to the k-mean classification of the different soils based on their physicochemical characteristics and land use type. The sampling sites for which soil properties were missing have not been included in the K mean classification and are presented as nd (not determined).

### Diversity and composition of the bacterial communities

**Figure 2:**
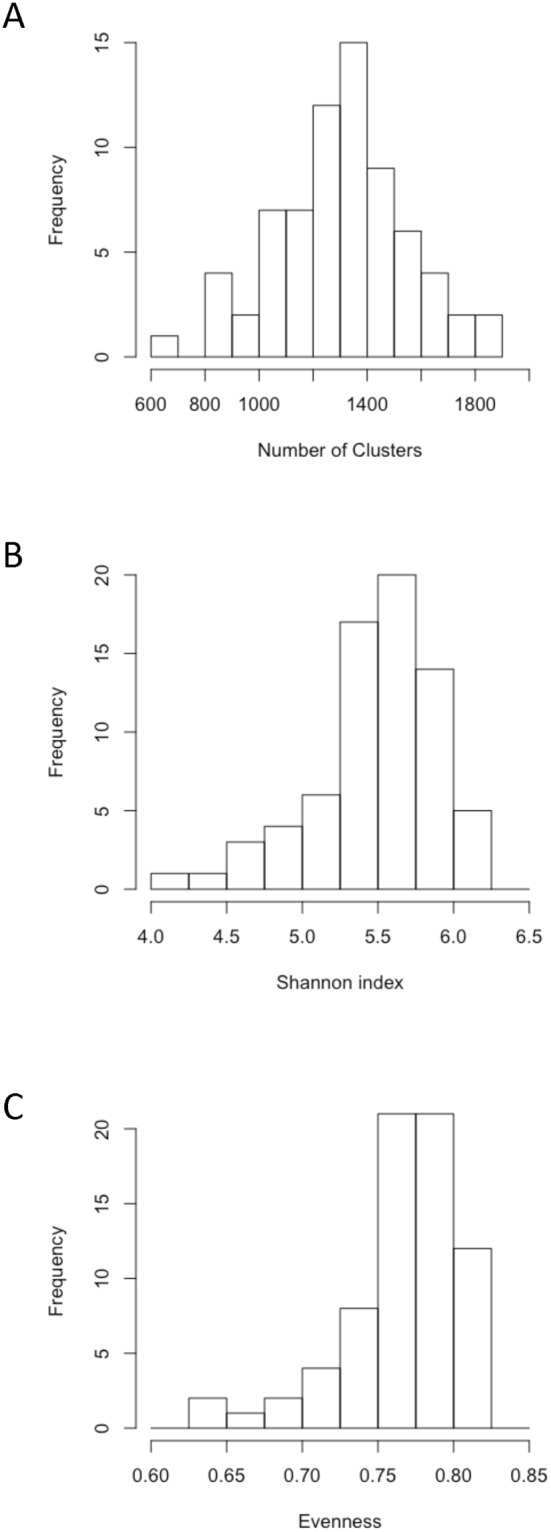
Relative distribution of bacterial richness (**A**), Shannon index (**B**) and evenness (**C**). Bacterial richness was determined from the number of clusters derived from the bioinformatics analysis.

We obtained more than 3*10^6^ 16S rRNA sequences from the 71 samples we collected, and a total of 8,085 sequences *per* soil sample after bioinformatics processing. The rarefaction curves of bacterial OTUs followed a logarithmic model (data not shown) without reaching a plateau. We detected a total of 34,190 OTUs among all the soil samples. At the European scale, the number of OTUs *per* soil sample ranged from 653 to 1,860 (mean: 1,307 OTUs; Standard Deviation (sd): 244; Fig. 2A). The Shannon index ranged from 4.1 to 6.2 (mean: 5.5; sd: 0.4; Fig. 2B), and evenness from 0.62 to 0.82 (mean: 0.76; sd: 0.04; Fig. 2C). The number of detected OTUs ranged from 1,022 to 1,860, 653 to 1,437, and 987 to 1,722 in soils dedicated to arable, forest, and grassland uses, respectively. The Shannon index varied from 4.1 to 5.6, 4.9 to 6.0, and 5.3 to 6.2, in forests, grasslands, and arable lands, and evenness from 0.63 to 0.78, 0.71 to 0.81, and 0.76 to 0.82. Altogether, the variations of richness, evenness, and of the Shannon index were in the range of those observed in the literature for a similar sequencing effort^29–32^. Nevertheless, these indices revealed a non-significant trend of lower bacterial richness and diversity from arable to forest soils. This is not in agreement with the literature since other studies demonstrated that microbial indices were affected by land-use at the plot scale^33^, the landscape scale^34^, and at more global scales^29,31,35^. The much greater variability of soil physicochemical properties relatively to land use in our study may account for this discrepancy since soil physicochemical characteristics are known to frequently be the most influencing factor of soil microbial indices, followed by land use^29,31,34,36^.

We detected a total of 27 phyla after taxonomic assignment (Supplementary Table 1). For each sample representing a specific environmental condition (*i.e*. a combination of a given soil property and a land use), the most represented bacterial phyla were Proteobacteria (average relative abundance 56.4%), Actinobacteria (15.7%), and Acidobacteria (7.1%) (Supplementary Table 1). The dominance of these phyla is in agreement with Lauber *et al*.^10^ and Delgado-Baquirezo *et al*.^30^. Other phyla, *i.e*. Bacteroidetes, Firmicutes, Planctomycetes, Chloroflexi, and Nitrospirae, were also represented (above 1% on average) but their relative abundance greatly varied according to the environmental conditions. We detected a total of 1,033 bacterial genera (data not shown), whose abundance varied according to environmental conditions from 157 genera in a Swedish forest soil, up to 397 in a Dutch arable soil. A total of 7.23% of the sequences remained unclassified at the genus level. The number of detected genera ranged from 310 to 397 in arable land (mean: 355; sd: 25), 157 to 313 in forest (mean: 260; sd: 48), and 264 to 386 in grassland (mean: 327; sd: 33), without any significant difference between land uses. These results are in the range of those reported in the literature^30,32,36^. The absence of a relationship between the number of genera and land use type may be related to the high number of genera represented at very low relative abundances, which we did not take in account. Similarly, Karimi *et al*.^36^ identified a total of 1,355 genera out of which only 47 had a relative abundance higher than 0.5%. Similar trends have also been observed at a global scale^30,32^.

### Spatial distance, soil properties and land use as drivers of bacterial communities

**Figure 3:**
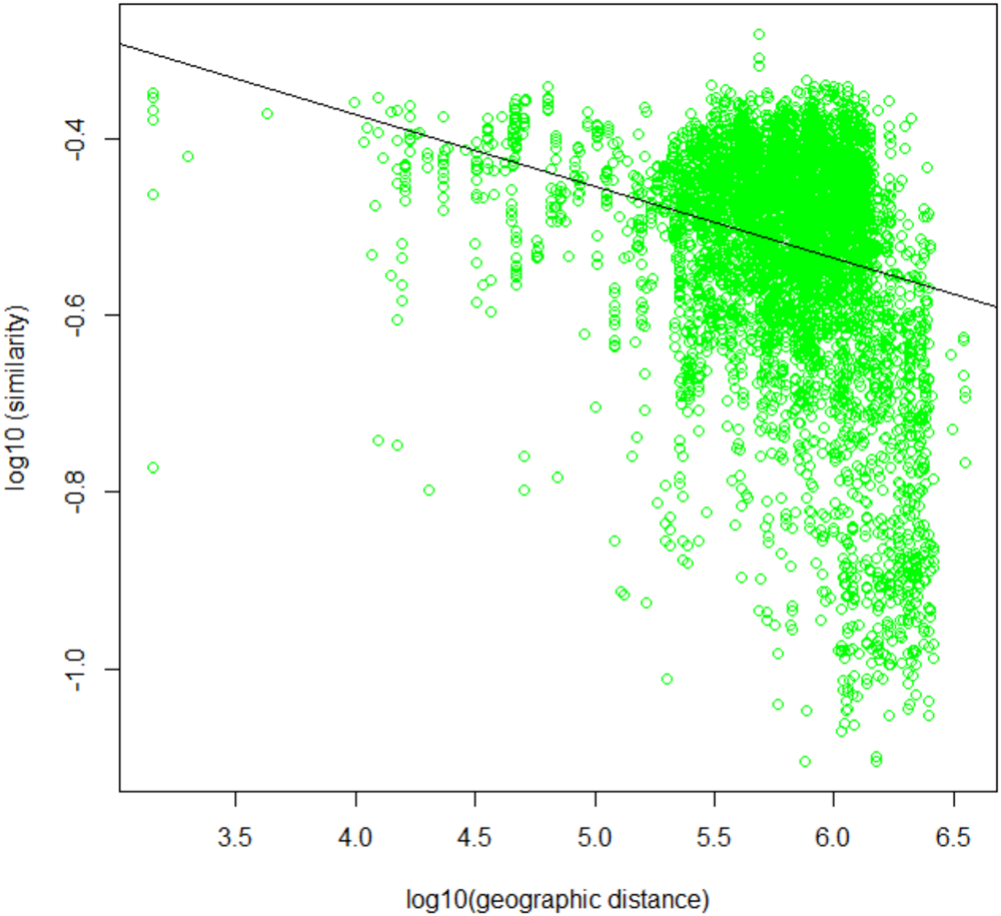
Taxa area relationship of soil bacterial community. Dots represent paired-comparison of sites for each distance class and line represents the weighted linear regression model used for the estimation of soil bacterial community turnover following the equation: ${\mathrm{log}}_{10}\,({{\rm{\chi }}}_{{\rm{d}}})=(\,\,-\,\,2\ast {\rm{z}})\ast {\mathrm{log}}_{10}\,({\rm{d}})+{\rm{b}}$. Estimated z: 0.0532; estimated b: −0.0034.

We performed further analyses to better evaluate the spatial structuration of soil bacterial communities and better specify the environmental and spatial drivers of the variations of bacterial communities across Europe. First, we evaluated TAR for soil bacterial communities at the European scale (Fig. 3). This relationship was significant since the similarity of bacterial community composition across sites significantly decreased with increasing geographical distance, as shown by a significant community composition turnover (z = 0.0532, b = −0.0034; r² = 0.07; P < 0.001). Although the turnover intensity was low, its level was in the range (0.1 to 0.23) of those reported in several studies applying the same methodology at different spatial scales and in different environmental matrices^37–42^. Furthermore, even lower turnover intensity values, ranging from 0.006 to 0.05, have recently been reported at the scale of France^12,13,43^. Altogether, the significant TAR observed at the scale of Europe in our study supports that soil bacterial community composition is non-randomly distributed and spatially structured. This result is in agreement with other studies focusing on soil microbial biogeography^12,44,45^. This spatial structuration of soil bacterial communities may be related to environmental filters (i.e. environmental selection) and to limited dispersal.

We applied two complementary approaches to better understand the factors involved in the spatial structuration of soil bacterial communities and supporting environmental selection and dispersal limitation. A descriptive approach based on NMDS applied on Unifrac distance (Supplementary Fig. 1) showed that pH (R = 0.87, *P* < 0.001, ANOSIM, 1,000 permutations), texture (R = 0.64, *P* < 0.001, ANOSIM, 1,000 permutations) and organic carbon content (R = 0.58, *P* < 0.001, ANOSIM) were major drivers of bacterial community structure. Other drivers such as the nitrogen content and CEC were strongly correlated to the organic carbon content; moreover, assimilable phosphorous was non significantly correlated to soil bacterial community variation (P > 0.24). Similarly, we recorded significant differences in the community structures of the three types of land use (grassland, arable, forest) (ANOSIM-land use: R = 0.32, *P* < 0.001). Even if soil properties and land use both had discriminating effects on soil bacterial communities, cross-effects must be considered together with the effect of distance between sites.

**Figure 4:**
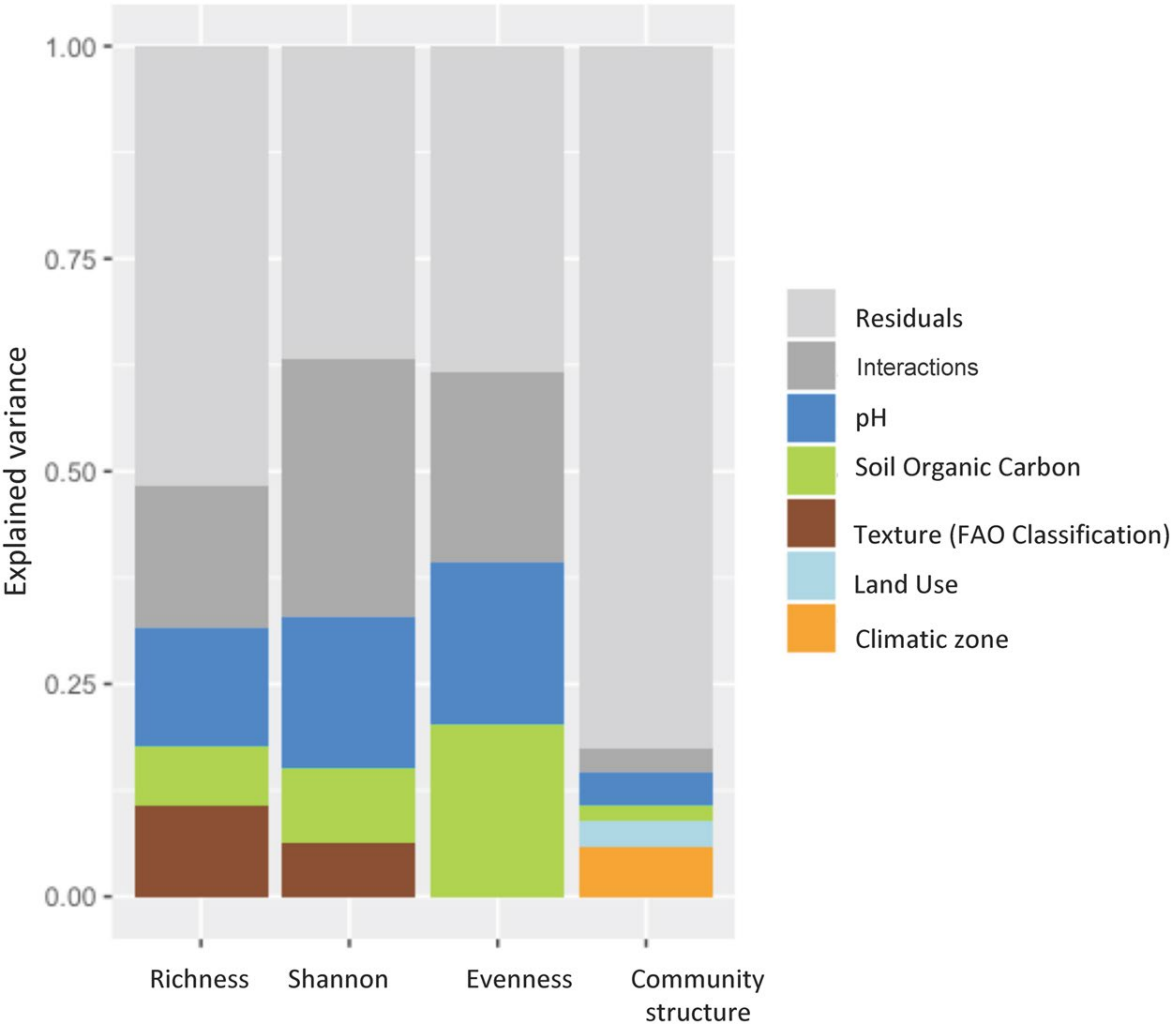
Variance partitioning of bacterial richness, diversity and evenness parameters within the European transect sampling.

To disentangle potential cross-effects and take the effect of distance between sites into account, we applied a parsimonious variance partitioning approach on the three diversity indexes (richness, Shannon index, and evenness) and on soil bacterial community structure to determine the relative importance of soil properties and/or land use and/or climate conditions and/or distance between sites on bacterial communities. This analysis notably showed that soil properties explained a large part of the variance of bacterial richness (48.4% of variance, *P* < 0.001); that effect was mostly related to pH, texture class and total carbon content, which accounted for 13.8%, 10.8%, and 6.9% of the variance of soil bacterial richness, respectively (Fig. 4). Other environmental parameters (land use, climate, and spatial autocorrelation) did not explain significant amounts of variance during model building, suggesting that they did not affect soil bacterial richness. We observed similar trends for the Shannon index and for evenness (63.2% and 61.7% of variance; respectively; *P* < 0.01) except that the texture was very slightly significant. The percentage of variance explained by the soil pH ranged from 17.7% for the Shannon index to 19.0% for evenness, while the total carbon content explained 8.6% for the Shannon index and 20.2% for evenness. Soil texture did not explain any variation of bacterial evenness and was slightly significant for bacterial Shannon index (6.4%, P < 0.1). For each diversity index, interactions between environmental factors represented a large portion of the explained variance (16.8% for richness, 30.4% for Shannon index, 22.5% for evenness). A large portion of the variance still remains unexplained, suggesting that other environmental factors may contribute to structure bacterial communities, and confirming previous findings^15,31,43^. Conversely to diversity indices, soil bacterial community structure based on Unifrac distance was only slightly explained by environmental factors (17.3% of variance, P < 0.001). Among all the environmental factors we identified, the soil pH (3.8%) ranked first, followed by land use (3.3%) and the total carbon content (1.7%). This is in agreement with other studies of soil microbial biogeography^7,11,14,31,43,46,47^ and is fairly related to the soil reactional conditions and trophic level and to biological interactions, e.g. between plants and soil bacteria. This study also identifies the climatic zone as a driving factor of soil bacterial community structure. Nevertheless, even if its marginal effect appeared to be substantial as compared to other variables, it was only very slightly significant (5.7%, P < 0.1). Its selection may be related to particular sites belonging to boreal and Mediterranean climatic zones located far from the rest of the transect. Surprisingly, longitude, latitude, or PCNMs (representing the neighbouring relationships between sites) were not selected when we built the model. This suggests that bacterial communities are not dispersal-limited at the scale of Europe. Nevertheless, other studies demonstrated that bacterial communities may be dispersal-limited at smaller spatial scales (France, Scotland)^12,13,44^. This discrepancy may be explained by differences in terms of sampling efforts.

### Soil clustering and indicator species analysis

Each sampling site exhibited different soil properties and land uses, so we further explored the relative contribution of these two types of parameters to bacterial diversity. For this purpose, we clustered sites according to the homogeneity of their soil physicochemical characteristics, land use and climate, as assessed by the k-mean method. The k-mean approach allowed us to the delineate four clusters with contrasting soil properties and covering different land uses: the first cluster (cluster 1, n = 27 sampling sites) included the three land uses (arable, grass, and forestry) and encompassed soils with a low carbon and nitrogen content, an average pH of *ca*. 6 and a coarse texture; the second cluster (cluster 2, n = 24) also encompassed representative soils from the three land uses but they displayed distinct properties, with medium carbon and nitrogen contents, an average pH of *ca*. 6 and a medium to fine texture; the third cluster (cluster 3, n = 5) included mainly forest soils but also one grassland, and the soils exhibited high carbon and nitrogen contents, an acidic pH and an organic texture; finally the fourth cluster (cluster 4, n = 18) included all three land uses but only one forest site and encompassed soils with a medium to fine texture, a neutral pH, and a low carbon content (Table 2). As suggested by Hermans *et al*.^48^ and Banerjee *et al*.^49^ specific soil bacterial genera may be identified as bioindicators of the different clusters. To test this hypothesis, we performed an indicator species analysis on the four clusters to determine whether specific genera were specifically associated with a single cluster, making them bioindicators. For each bacterial genus identified in this analysis, the relative abundance and the relative coverage of a cluster condition are presented in Table 2. For example, the *Blastochloris* genus, whose maximum abundance reached 18.6%, is represented in 76% of the samples of cluster 3, while the *Pseudolabrys* genus, whose maximum abundance reached 4.2%, is represented in 50% of the samples of cluster 4. Previous reports have shown that this approach can be successfully applied to identify microbial groups (*i.e*. fungi or bacteria), phyla, or even genera as environmental indicators^50,51^. We recorded a total of 118 genera with an average relative abundance above 0.1% (Table 2). Cluster 1 was characterised by 17 indicator genera, mainly related to the Proteobacteria (11) or Firmicutes (3) phyla; the most abundant indicator genera were assigned to *Pseudolabrys* (max. relative abundance 4.23%) and *Azospira* (4.11%). Cluster 2 harboured only three indicator genera, belonging to the Elusimicrobia and Proteobacteria phyla and assigned to the *Elusimicrobium* (2.61%)*, Pelobacter* (0.41%) *and Xanthomonas* (0.27%) genera. Cluster 3 was characterised by 59 indicator genera mainly related to Proteobacteria, Actinobacteria, and Bacteroidetes; the most abundant indicator genera were assigned to *Blastochloris* (max. relative abundance 18.65%), *Halophaga* (13.2%), and *Steroidobacter* (8.51%). Nevertheless, this result remains to be confirmed by other studies because of it was obtained from a small number of sites (n = 5). Cluster 4 encompassed 39 indicator genera mostly related to Proteobacteria and Actinobacteria; the most abundant indicator genera were assigned to *Gaiella* (max. relative abundance 9.65%) and *Bacillus* (5.22%). Overall, we found higher numbers of indicator genera in the clusters with the lowest variety of land uses (clusters 3 and 4), and conversely lower numbers of indicator genera in the clusters encompassing more different land uses (clusters 1 and 2), suggesting that land use is a major driver of soil biodiversity^52^.

**Table 2:** Identification of bacterial genera associated to a particular environment.

Phylum or class	Genus	Relative abundance (%)			Relative contribution of soil properties, land use, and climate on the explanation of the variance (%)							Soil condition clusters			
					Soil properties					land use	climate				
		min	med	max	total	pH	texture	total C	total N			1	2	3	4
Acidobacteria	Acidobacterium	0	0	0.19										0.55**	
	Candidatus_Koribacter	0	0.01	0.53										0.42*	
	Holophaga	2.82	6.02	13.2						15.22				0.33**	
Actinobacteria	Acidothermus	0.01	0.32	5.75	41.08	41.08				5.79				0.48*	
	Actinoalloteichus	0	0	0.12											0.57*
	Actinospica	0	0	0.33	6.13	6.13				23.91				0.7***	
	Cryobacterium	0	0.05	0.31											0.42*
	Ferrimicrobium	0	0.06	0.24	34.47	10.03		18.45	5.99		15.3				0.36*
	Ferrithrix	0	0.01	0.31	16.28	16.28					7			0.59**	
	Frigoribacterium	0	0.29	8.51	29.94		29.94							0.73***	
	Gaiella	0.01	2.81	9.65	45.79			29.09	16.7						0.37**
	Geodermatophilus	0	0	0.56											0.8*
	Humicoccus	0	0.14	1.46	24.04			24.04				0.44*			
	Marmoricola	0	0.17	1.18	64.86	14.4		31.68	18.78						0.47**
	Microlunatus	0	0.04	0.4	17.56	17.56									0.51*
	Mycobacterium	0.02	0.33	2.61										0.47**	
	Nocardioides	0	0.58	2.04	55.4			37.64	17.76						0.44***
	Patulibacter	0.04	0.3	1.52	28.89	28.89					6.24			0.41*	
	Rhodococcus	0	0.07	0.84											0.45*
	Solirubrobacter	0.01	0.62	3.39	38.78	25.9		12.88		10.14					0.49***
	Streptacidiphilus	0	0	0.17										0.77***	
	Streptomyces	0	0.27	0.73	46.08	33.92		12.16			6.12				0.43**
Armatimonadetes	Chthonomonas	0	0.02	0.37	20.8		20.8				9.91			0.6***	
Bacteroidetes	Barnesiella	0	0.02	1.05										0.72**	
	Chitinophaga	0	0.11	0.69										0.46**	
	Ferruginibacter	0.01	1.09	3.01	10.04	4.69		5.35		28.68	4.62	0.35*			
	Flavisolibacter	0	0.25	1.36											0.45**
	Mucilaginibacter	0	0.01	0.36										0.65**	
	Segetibacter	0	0	0.25							15.77				0.71*
	Solitalea	0	0.19	0.96	9.73	9.73								0.46**	
Chloroflexi	Caldilinea	0	0.1	0.31	67.59	22.77		35.97	8.85						0.42***
	Dehalococcoides	0	0.02	0.27						16.85					0.46*
	Roseiflexus	0	0.61	3.69	24.83	14.23		10.6		68.55	6.62				0.5***
	Thermomicrobium	0	0.14	1.32	51.51	33.46		18.05			5.5				0.53**
Deinococcus-Thermus	Deinococcus	0	0	0.69										0.97**	
Elusimicrobia	Elusimicrobium	0	0.09	0.41						10.09			0.41**		
Firmicutes	Acetobacterium	0	0.01	0.71	19.89		19.89					0.6*			
	Ammoniphilus	0	0	0.26	10.32			5.84	4.48	11.11	14.26	0.6*			
	Bacillus	0	1.55	5.22	26.94	4.9		7.89	14.15	39.05	3.91				0.37*
	Butyrivibrio	0	0	0.35	24.27	24.27								0.89***	
	Moorella	0	0.03	0.21	23.55	7.06		16.49							0.46*
	Solibacillus	0	0.02	0.43	26.48	4.94		21.54				0.54*			
	Syntrophothermus	0	0.01	0.15	12.77			12.77							0.53*
	Thermacetogenium	0	0.02	0.14						30.78					0.49*
	Thermincola	0	0.02	0.26						15.76					0.51**
Gemmatimonadetes	Gemmatimonas	0	0.76	3.09	55.13		55.13			9.9					0.42**
Nitrospirae	Leptospirillum	0	0.06	0.47	45.72		45.72							0.53**	
	Nitrospira	0	0.6	2.73	48.64	30.48		18.16			5.81				0.43**
Planctomycetes	Isosphaera	0	0.04	1.55	37.37	37.37								0.69**	
	Planctomyces	0.05	0.58	1.77	9.64	9.64					14.09	0.34*			
	Schlesneria	0	0.14	1.72										0.63***	
	Singulisphaera	0	0.13	0.8										0.41*	
Proteobacteria	Acidicaldus	0	0.2	3.51	34.3	34.3								0.58**	
(alpha)	Acidisoma	0	0.03	1.26										0.69***	
	Acidisphaera	0	0.04	1.93	38.46	38.46					13.08			0.68***	
	Acidocella	0	0.01	1.3	26.55	11.98	14.57							0.83***	
	Altererythrobacter	0	0.1	0.42	51.71	36.49		15.22			2.92				0.41*
	Asticcacaulis	0	0.01	0.16	6.64	6.64								0.52**	
	Bauldia	0	0.05	0.31	25.4		25.4				6.97	0.43*			
	Beijerinckia	0	0.04	1.9	51.17		42.02		9.15					0.77***	
	Blastochloris	0	0.2	18.65										0.76***	
	Bosea	0	0.21	2.66											0.47*
	Caulobacter	0.02	0.27	0.74							25.62			0.38**	
	Chelatococcus	0	0.02	0.33	58.88	58.88					5.56				0.44*
	Devosia	0.01	0.7	3.3						36.78	9.02	0.39*			
	Erythrobacter	0	0.01	0.15	5.38			5.38		18.99					0.43*
	Gluconacetobacter	0	0	0.25	17.31		17.31				25.62			0.65**	
	Inquilinus	0	0.01	0.59										0.79***	
	Methylobacterium	0	0.04	0.77						33.43					0.54*
	Methylocapsa	0	0	0.1	25.59		25.59			9.13				0.81***	
	Methylosinus	0	0.11	2.03	34.18	12.54			21.64		10.02				0.51*
	Methylovirgula	0	0	0.41										0.86**	
	Microvirga	0	0.11	2.02	39.44	39.44									0.52*
	Novispirillum	0	0	0.28						19.97				0.8***	
	Novosphingobium	0.01	0.14	0.53	7.33			7.33			16.38			0.38*	
	Ochrobactrum	0	0.14	0.62	12.39	12.39					24.91	0.38*			
	Paracraurococcus	0	0	0.15											0.9*
	Phenylobacterium	0.1	0.72	6.15	45.01	45.01								0.56***	
	Pseudolabrys	0.02	0.54	4.23	46.65	11.1	35.55					0.5**			
	Rhodovastum	0	0.02	0.57										0.49*	
	Roseobacter	0	0.01	0.12										0.57***	
	Roseomonas	0	0.04	0.17											0.44**
	Shinella	0	0.07	0.4	52.87	26.77		17.74	8.36			0.39*			
	Skermanella	0.06	0.41	2.08						25.63				0.38*	
	Sphingomonas	0	0.27	1.4	36.97	36.97				8.54					0.43**
	Telmatospirillum	0	0	0.15										0.91***	
	Thalassospira	0	0.03	0.3							12.41			0.61***	
Proteobacteria	Achromobacter	0.02	0.85	3.72	26.57			11.69	14.88			0.38*			
(beta)	Azospira	0.06	1.57	4.11	36.01			23.72	12.29			0.34*			
	Burkholderia	0.51	1.57	4.98	6.69				6.69		14.92			0.34*	
	Collimonas	0	0	0.32										0.59*	
	Dokdonella	0	0.22	1.56	46.07			27.92	18.15			0.45*			
	Duganella	0	0.01	0.46	32.95	24.19		8.76				0.51*			
	Georgfuchsia	0	0	0.32								0.77*			
	Herbaspirillum	0	0.05	0.49	13.11			13.11							0.44*
	Herminiimonas	0	0.02	0.35						19.55				0.49*	
	Methylotenera	0	0.05	1.99										0.54*	
	Nitrosospira	0	0.05	0.36						27.28		0.43*			
	Pandoraea	0	0	0.82										0.9***	
	Ramlibacter	0	0.06	0.68	16.7			16.7							0.55**
	Sphaerotilus	0	0	0.47										0.55*	
	Variovorax	0	0.43	1.16	8.5	8.5									0.34*
Proteobacteria	Aquicella	0.17	0.79	4.5						14.08				0.5***	
(gamma)	Coxiella	0.01	0.13	0.57	7.58			7.58						0.48***	
	Cycloclasticus	0	0.01	0.31	10.72			10.72						0.5*	
	Diplorickettsia	0	0.01	0.19										0.68***	
	Dyella	0	0.01	0.85										0.75*	
	Halomonas	0	0.12	1.01							16.31			0.52***	
	Legionella	0	0.02	0.17										0.53***	
	Nevskia	0	0	0.17	5.26	5.26				21.51				0.65**	
	Rhodanobacter	0.05	0.51	4.96	56.07	52.74		3.33			8.58			0.58***	
	Steroidobacter	0.67	2.9	12.6						20.01				0.46**	
	Xanthomonas	0	0.45	2.61	32.71	32.71							0.43*		
Proteobacteria	Geothermobacter	0	0.05	0.21										0.37*	
(delta)	Hyalangium	0	0.01	0.14											0.49*
	Myxococcus	0	0	0.1											0.6*
	Pelobacter	0	0.06	0.27	52.22	17.03	35.19						0.4*		
Verrucomicrobia	Opitutus	0	0.12	1.69										0.55**	

All the transect site characteristics (physical, chemical, geographical, climatic and land use data) were used to organize the sites into four soil condition clusters (cluster 1: low C and N contents; cluster 2: moderately rich soils; cluster 3: nutrient-rich and acidic soils; cluster 4: Low C and N contents and high pH values) using the k-means method. An indicator species analysis was performed in each of these clusters to identify the bacterial genera preferentially associated with one of these specific clusters. For each genus significantly associated with a cluster, its sensitivity (*i.e*. the probability of finding the genus in sites belonging to the surveyed environment) is reported, with stars denoting the significance level of the indicator species analysis (***P < 0.001; **P < 0.01; *P < 0.05). For each genus significantly associated with one cluster, minimum, median and maximum relative abundances are presented. Models predicting the abundance of each genus were built and led to assess the amount of variance explained by environmental factors (soil properties, land use, climate).

The bioindicator analysis also revealed that such an analysis must be conducted at a fine taxonomic level (*i.e*., the genus level). We indeed demonstrated that different indicator genera belonging to a same phylum could be indicators of different soil conditions (*i.e*. cluster type). For example, within the Actinobacteria phylum, the *Actinospica* genus was an indicator of cluster 3 and the *Geodermatophilus* genus an indicator of cluster 4 (Table 2). Another important feature is related to the relative abundance of these indicator genera. Indicators were represented at high or low relative abundance levels (maximum relative abundance above 10% and below 0.5%, respectively) *e.g*. the *Blastochloris, Holophaga* or *Steroidobacter* genera, and *Myxococcus* or *Collimonas*, respectively. The latter would be good candidates as indicators of specific environmental conditions. The *Collimonas* genus was indeed more specifically found in cluster 3 (forest) confirming previous works done on this genus^18,25^. The *Myxococcus* genus was more specifically found in cluster 4 (arable land: 11, grassland: 4). Then we constructed models to refine and predict the contribution of the three environmental parameters - soil properties, land use, and climate - associated with these clusters for each indicator genus (Table 2). For example, the indicator genus *Acidothermus* in cluster 3 was mostly explained by soil pH (41.08%) and land use (5.79%), while the indicator genus *Gaiella* in cluster 4 was explained by the total carbon (29.09%) and nitrogen (16.7%) contents. Globally, a broad range of the bioindicators (40 out of 118) identified in this study were determined by the soil pH, confirming the importance of this parameter for soil biodiversity as reported in previous studies^7,9,11,14^, while C content^50^, climate zone, land use^15^ and texture^13^ explained the relative distribution of 30, 27, 25 and 12 other bioindicators, respectively. Only 12 bioindicator genera were explained specifically by land use exclusive of any other parameter (Table 2): in cluster 1, *Nitrospira* (Betaproteobacteria); in cluster 2, *Elusimicrobium* (Elusimicrobia); in cluster 3, *Skermanella* (Alphaproteobacteria), *Steroidobacter* (Gammaproteobacteria), *Novispirillum* (Alphaproteobacteria), *Herminiimonas* (Betaproteobacteria), *Holophaga* (Acidobacteria), and *Aquicella* (Gammaproteobacteria); in cluster 4, *Methylobacterium* (Alphaproteobacteria), *Thermoacetogenium* (Firmicutes), *Dehalococcoides* (Chloroflexi), and *Thermincola* (Firmicutes). Nevertheless, 37 of the bacterial genera identified as indicators of clusters remained unexplained by any of the parameters tested (soil properties, land use, climate), *e.g. Blastochloris* (max. relative abundance of 18.65%) or *Mycobacterium* (max. relative abundance of 2.61%), *Myxococcus* (0.1%), or *Collimonas* (0.32%).

### Land use effects within soil clusters

**Figure 5:**
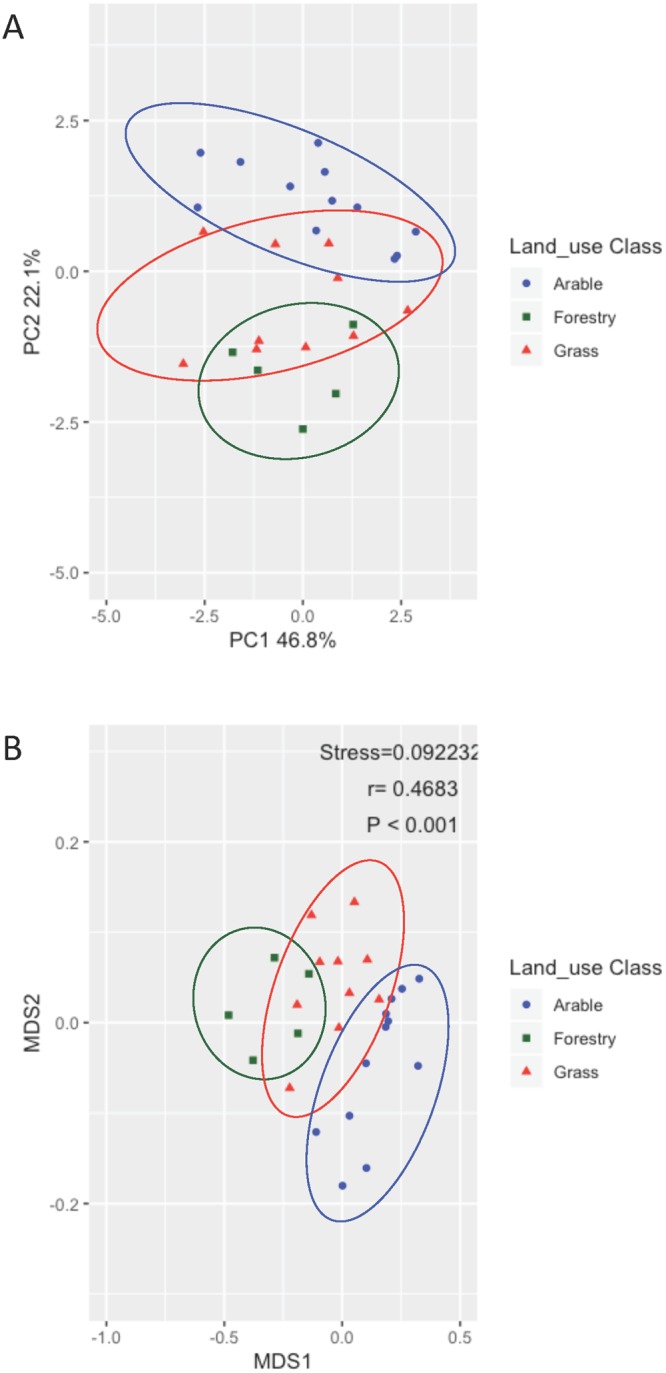
Multivariate analysis of soil physicochemical properties (**A**) and bacterial communities (**B**) according to the land use within soil cluster 1. The correspondence factor analyses generated from the physicochemical characteristics (**A**) or relative abundance of all the detected taxa (**B**) in the soils belonging to cluster 1 illustrate the impact of land use on bacterial communities. Colored ellipses corresponding to the different land uses have been manually added.

We further tested the relative impacts of land use on the bacterial communities under similar soil conditions using the land use distribution found in each cluster as generated by the k-mean classification. In contrast with the very small cluster 3 including only five sites (4 forests and 1 grassland) and cluster 4 that included only one forest site out of 18, clusters 1 and 2 encompassed the three land uses (*i.e*. forestry, arable land, and grassland). We therefore analysed clusters 1 and 2 to test whether variations of bacterial communities were mostly explained by land use or soil properties. We performed multivariate analyses independently, on the soil physicochemical properties and then on the distribution of the bacterial genera characterising the sites encompassed in clusters 1 and 2. The results showed that the soils were mostly grouped in terms of soil properties according to the land use, with a clear separation between forests and other land uses as confirmed by ANOSIM analysis (in cluster 1: R_soil data_ = 0.60, *P* < 0.001; in cluster 2: R_soil data_ = 0.68, *P* < 0.001) (Fig. 5). Furthermore, bacterial genera appeared to be distributed differentially in cluster 1 according to the land use, not to the soil properties (ANOSIM analysis R_genus data_ = 0.524, *P* < 0.001); the major driving effect of the land use in this cluster was also supported by the absence of a significant correlation (*P* > 0.05) between soil parameters and the distribution of bacterial genera (Fig. 5). This was not the case in cluster 2 (ANOSIM analysis R_genus data_ = 0.251, p = 0.055) that encompassed forest sites differing by their constitutive soil properties. Taken together, our data show that, under similar soil properties, land use is a major driver of bacterial community structure. Compared with previous reports^6,8,53,54^, this conclusion is strongly supported by the large variety of soil properties for each land use in contrast with previous studies that addressed the land use effect only on a given soil type^54–56^.

## Conclusions

We analysed the effect of soil properties and land use on the soil bacterial communities using an unprecedented extensive European transect allowing us to sample soils representative of a range of soil properties (organic matter content, pH, texture) from different geographical and climatic zones across Europe. Our analysis generated a broad overview of the soil bacterial diversity, richness and composition across Europe, which is strongly complementary to other intensive approaches dedicated to specific countries in Europe (the United Kingdom^11^, France^31,36^ and the Netherlands). Combining soil analyses with 16S rRNA gene amplicon pyrosequencing revealed that the soil bacterial communities are non-randomly distributed across Europe but determined by environmental factors. More specifically, soil properties, notably the pH and texture, appeared to be the main drivers of soil bacterial community composition and distribution at the European scale. However, under given soil properties corresponding to delineated clusters, the influence of land use was apparent. We identified bacterial genus indicators of soil physicochemical properties through a cluster analysis; some genera were land use specific within these clusters.

Taken together our data provide an overview of the soil bacterial diversity across Europe and identify potential bacterial indicators of different soils and land uses. We confirmed at a large spatial (continental) scale the strong impact of soil properties on soil bacterial communities, but we also showed that under similar soil conditions, land use also contributed to their structure. These findings and the identification of indicators open up onto stimulating new prospects in terms of land management for monitoring soil biodiversity and ultimately expected services from soils.

## Methods

### Site description, soil properties and sampling strategy

**Figure 6:**
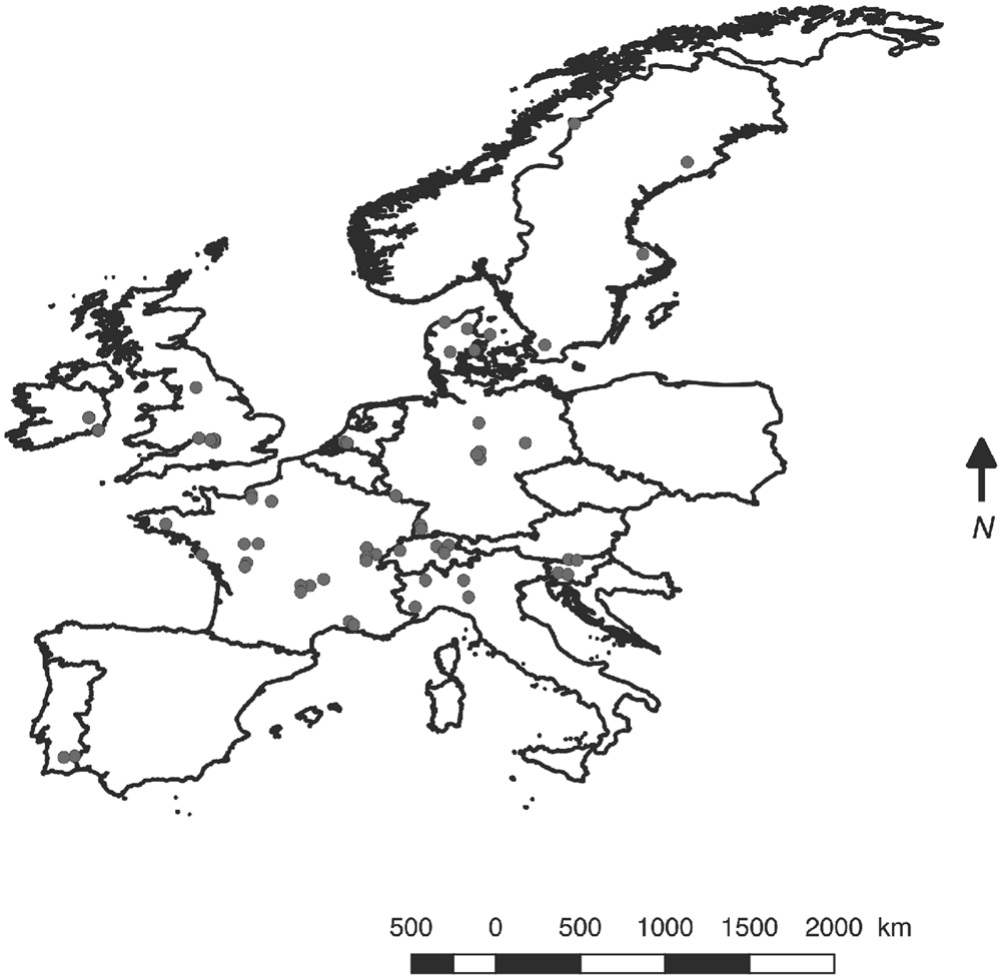
Mapping of sampling sites across the European transect. Individual sampling sites are shown in black. Geographical coordinates were degraded at the 1 km^2^ scale for privacy reasons.

Soil samples were collected across 71 European sites (Fig. 6) in 11 countries (5 in Denmark, 19 in France, 9 in Germany, 6 in Ireland, 4 in Italy, 2 in The Netherlands, 2 in Portugal, 4 in Slovenia, 4 in Sweden, 5 in Switzerland, and 11 in the United Kingdom), as described in Stone *et al*.^28^. The sampling sites were identified on the basis of EFSA spatial legacy data (Version 1.0) provided by the Joint Research Centre, of the European Commission^57^. We chose four parameter maps among the 52 spatial layers available: topsoil organic matter; topsoil pH in water; topsoil texture class; and EFSA Corine land cover data. The sites encompassed three types of land uses (arable, forest, grassland) (see Table 1 for details). Soil sampling was performed from early September to mid-November 2012 to avoid the influence of seasonal variation using the same standardized optimised procedure (SOP)^28^. For each site, a composite sample was made from twelve cores (5 cm in diameter and 5 cm depth) randomly taken within a 1 × 1 m surface. All soil samples were sieved to <2 mm and prepared for soil analyses by Teagasc and DNA extraction was performed in Dijon by the GenoSol platform, UMR Agroécologie, https://www2.dijon.inra.fr/plateforme_genosol/plateforme-genosol. Soil properties were measured as described in Creamer *et al*.^58^. Briefly, soil texture was characterised following the particle pipette size method^59^. Total C and N were measured according to the ISO 10694 standard^60^; organic carbon (OC) was determined by LECO elemental analysis, conducted on 0.25 mm dry milled soil sub-samples^61^. The cation exchange capacity (CEC) was analysed using the BaCl_2_ extraction method^62^. The pH was measured in a 1:2.5 soil in water suspension using a glass electrode^63^. N mineralisation was analysed using the Illinois soil nitrogen test for amino sugar-N^64^. Available phosphorus content was measured using the Mehlich 3 methodology^65^. The sites were grouped based on their pH values (<5, between 5 and 7, >7), organic C content (less than 2%, between 2 and 15%, over 15%) and texture (fine, medium or coarse). The distribution and characteristics of the samples are presented in Table 1 and Fig. 1.

### Molecular characterisation of soil bacterial communities

DNA was extracted and purified from all soil samples using the improved ISO 11063 soil DNA extraction procedure as described by Plassart *et al*.^66^. Briefly, 1 g of soil of each soil sample was added to a 15 ml Falcon tube containing 2.5 g of 1.4 mm diameter ceramic beads, 2 g of 100 μm diameter silica beads and 4 mm diameter glass beads. The samples were then mixed with a solution of 100 mM Tris-HCl (pH 8), 100 mM EDTA (pH 8), 100 mM NaCl, 2% (w/v) of polyvinylpyrrolidone (40 g mol^−1^) and 2% (w/v) sodium dodecyl sulphate. The tubes were shaken for 3 × 30 s at 4 m.s^−1^ in a FastPrep®−24 (MP-Biomedicals, NY, USA), before incubation for 10 min at 70 °C and centrifugation at 14,000 x g for 1 min. The supernatant was removed and then proteins were precipitated with 1/10 volume of 3 M sodium acetate prior to centrifugation (14,000 g for 5 min at 4 °C). Nucleic acids were precipitated by adding 1 volume of ice-cold isopropanol. The DNA pellets obtained after centrifugation (14,000 g for 5 min at 4 °C) were washed with 70% ethanol. Crude DNA extracts were then purified using a MinElute gel extraction kit (Qiagen, France) and quantified using a QuantiFluor staining kit (Promega, USA), prior to further investigations.

The diversity, composition and structure of the bacterial communities were determined on the 71 soil DNA samples by 16S rRNA gene amplicon pyrosequencing. A 450 bp fragment of the 16S rRNA gene was amplified using the primers F479 (5′- AGCMGCYGCNGTAANAC-3′) and R888 (5′-CCGYCAATTCMTTTRAGT-3′)^43^. Five ng of DNA were used in 25 μL of PCR mixture. The PCR was conducted under the following conditions: 94 °C for 2 min, 35 cycles of 30 s at 94 °C, 30 s at 52 °C, and 1 min at 72 °C, followed by 7 min at 72 °C. The PCR products were purified using a MinElute gel extraction kit (Qiagen, Courtaboeuf, France) and quantified using the PicoGreen staining Kit (Molecular Probes, Paris, France) on an Infinite 200 Pro fluorimeter (Tecan, Lyon, France). A second PCR of nine cycles was then conducted for each sample under similar PCR conditions using purified PCR products as DNA template and modified F479 and R888 primers containing 10 bp multiplex identifiers added at 5′ position to specifically identify each DNA sample. Two 25 µL reactions were performed for each DNA sample and pooled, before purification and quantification as previously described. Pyrosequencing was then carried out on a GS FLX Titanium (Roche 454 Sequencing System) by Genoscreen (Lille, France).

We performed the 16S rRNA gene sequence analysis according to Terrat *et al*.^43^, using the GnS-PIPE^67^. Briefly, all the raw reads were sorted according to the multiplex identifier sequences. The raw reads were then quality filtered and trimmed based on (i) their length (370 b), (ii) their number of ambiguities (Ns = 0) and (iii) their primer(s) sequence(s). The reads were dereplicated (*i.e*. clustering of strictly identical sequences) and aligned using INFERNAL alignment tool^66^, then clustered into OTUs using a PERL program that groups rare reads to abundant ones, and does not count differences in homopolymer lengths^67^. A filtering step was then carried out to check all singletons based on the quality of their taxonomic assignments to keep or discard them from the data sets. Finally, the retained reads were homogenised by random selection to 8,085 sequences *per* sample (the minimum number of reads per sample after GnS-PIPE processing) to compare the datasets and avoid biaised community comparisons. These sequences were then clustered into OTUs (97% similarity threshold) prior to determining diversity and richness indices. OTUs were further identified taxonomically using the Silva database. The raw data sets are available on the European Bioinformatics Institute database system under BioProject id. 254033, accession numbers from SAMN04384084 to SAMN04384190.

### Statistical analyses

All the statistical analyses were performed using R free software version 3.0.2^68^, using the packages Ade-4^69^, Vegan^70^ and Indicspecies^71^. The physical and chemical heterogeneity of soils was assessed by Hill & Smith Principal Component Analysis for mixed data using the dudi.hillsmith function in ade4 package. This analysis involved the following set of variables: Soil texture according to the FAO classification (qualitative variable), soil organic carbon, total nitrogen content, available phosphorous, soil pH, and cation exchange capacity. Land use was used as a categorical variable, and differences among land uses were tested by permutation (Monte-Carlo test, 1,000 permutations).

The turnover rates (z) for bacterial community composition were derived from the slope of the Taxa-Area Relationship (TAR) as described in Ranjard *et al*.^12^ following the method described in Harte *et al*.^72^ and by applying the equation:

$${\mathrm{log}}_{10}\,({{\rm{\chi }}}_{{\rm{d}}})=(\,\,-\,\,2\ast {\rm{z}})\ast {\mathrm{log}}_{10}\,({\rm{d}})+{\rm{b}}$$

where χ_d_ is the observed Sørensen’s similarity between two soil samples that are d meters apart from each other. χ_d_ and d are determined based on community structure data and geographical coordinates of sampling sites; respectively. b is the intercept of the linear relationship and z the turnover rate of the community composition. The z estimate and its 95% confidence interval were derived from the slope (−2*z) of the relationship between similarity and distance by weighted linear regression. Only distances greater than 1 km were considered.

Differences in soil microbial community structure were characterized using UniFrac distances^73^. Non metric multidimensional scaling (NMDS) was then used to graphically depict differences between soil microbial communities using the MetaMDS function of the vegan package. The significance of the observed clustering of samples on the ordination plot was assessed by an analysis of similarity (ANOSIM, 1000 permutations).

A variance partitioning approach was used to investigate the relative influences of soil physicochemical characteristics (FAO classification for soil texture, total C content, assimilable P, soil pH, and CEC), land use, climatic zone, and geographical location (represented by geographical coordinates and spatial descriptors derived from geographical coordinates by means of a principal coordinates neighbour matrices approach, PCNMs, representing neighbourhood relationships between sites^69^) on diversity indices (richness, evenness, and Shannon-Weaver index) and on soil bacterial community structure. Total nitrogen was not considered as an explanatory variable because it was highly correlated to the total soil carbon content (r^2^ = 0.96). This approach consisted of two successive steps. First, we investigated the effect of environmental variables. Then, we tested the effect of geographical location and neighbourhood relationships on the residuals derived from the first step using the same methodology. For diversity indices, each step first consisted in evaluating each combination of explanatory variables relatively to its adjusted R^2^ (to maximize) and its Bayesian Indication Criterion (to minimize) using the regsubsets function in the leaps package. This led to a reduced set of explanatory variables which was submitted to an iterative selection by means of Canonical Redundancy Analysis using the rda and ordiR2Step functions in the vegan package (forward selection) to identify the most parsimonious model. For bacterial community structure, each step was based on a distance-based redundancy analysis on the Unifrac distance matrix and consisted of a forward selection of the most parsimonious model by the capscale and ordiR2step functions in the vegan package. Finally, in each case, the total explained variance and the marginal effect of the selected explanatory variables were determined through an ANOVA-like approach using the anova.cca function in the vegan package.

In order to evaluate if microbial groups could represent indicators of soil conditions, land use and/or climate conditions, we clustered the sites into different environment types using the k-means method^74^. The k-means method is one of the most widely used techniques to establish clusters from environmental observations. First, initial groups were formed, and centroids were calculated as barycenters of the clusters. Then, the algorithm assigned each observation for the group to the closest centroid, and new centroids were calculated. These steps were repeated until the centroids no longer moved. The data used for this classification were soil physicochemical properties (pH, texture, organic C, total N) and land use (arable, forest, grassland). This classification method identified four clusters (cluster 1: nutrient poor soils, cluster 2: moderately rich soils, cluster 3: nutrient-rich and acidic soils, and cluster 4: nutrient-poor soils with an alkaline pH). To identify the indicator genera of each cluster obtained by the k-mean method, the multipatt function of the indicspecies package was used^71^. Based on the microbial groups identified as environmental indicators, a variance partitioning approach was used to model the influence of soil physicochemical properties, land use, climate and geographic location on the relative abundance of each bacterial genus. The land use effect within the clusters was tested by multivariate analysis and analysis of similarity (ANOSIM).

## Supplementary information

Supplementary Information

## References

[1] Lemanceau P (2015). Understanding and managing soil biodiversity: a major challenge in agroecology. Agron. Sustain. Dev..

[2] Bender SF (2016). An underground revolution: biodiversity and soil ecological engineering for agricultural sustainability. Trends Ecol. Evolut..

[3] Ranjard L (2010). Biogeography of soil microbial communities: a review and a description of the ongoing French national initiative. Agron. Sustain. Dev..

[4] Latour X (1996). The composition of fluorescent pseudomonad populations associated with roots is influenced by plant and soil type. Appl. Environ. Microbiol..

[5] Berg G, Smalla K (2009). Plant species and soil type cooperatively shape the structure and function of microbial communities in the rhizosphere. FEMS Microbiol. Ecol..

[6] Kuramae E (2011). Soil and plant factors driving the community of soil-borne microorganisms across chronosequences of secondary succession of chalk grasslands with a neutral pH. FEMS Microbiol. Ecol..

[7] Fierer N, Jackson RB (2006). The diversity and biogeography of soil bacterial communities. Proc. Natl. Acad. Sci. USA.

[8] Lauber CL (2008). The influence of soil properties on the structure of bacterial and fungal communities across land-use types. Soil. Biol. Biochem..

[9] Lauber CL (2009). Pyrosequencing-based assessment of soil pH as a predictor of soil bacterial community structure at the continental scale. Appl. Environ. Microbiol..

[10] Lauber CL (2013). Temporal variability in soil microbial communities across land-use types. ISME J..

[11] Griffiths RI (2011). The bacterial biogeography of British soils. Environ. Microbiol..

[12] Ranjard L (2013). Turnover of soil bacterial diversity driven by wide-scale environmental heterogeneity. Nat. Commun..

[13] Chemidlin Prévost-Bouré N (2014). Similar processes but different environmental filters for soil bacterial and fungal community composition turnover on a broad spatial scale. Plos One.

[14] Jeanbille M (2015). Soil parameters drive the structure, diversity and metabolic potentials of the bacterial communities across temperate beech forest soil sequences. Microb. Ecol..

[15] Thomson BC (2015). Soil conditions and land use intensification effects on soil microbial communities across a range of European field sites. Soil. Biol. Biochem..

[16] Marschner P (2001). Soil and plant specific effects on bacterial community composition in the rhizosphere. Soil Biol. Biochem..

[17] Mougel C (2006). Dynamic of the genetic structure of bacterial and fungal communities at different developmental stages of *Medicago truncatula* Gaertn. cv. Jemalong line J5. New Phytol..

[18] Uroz, S. *et al*. Specific impacts of beech and Norway spruce on the structure and diversity of the rhizosphere and soil microbial communities. *Sci. Rep*. **6**, 10.1038/srep27756 (2016). 10.1038/srep27756 PMC4908602 27302652

[19] Lundberg DS (2012). Defining the core *Arabidopsis thaliana* root microbiome. Nature.

[20] Mendes R, Garbeva P, Raaijmakers JM (2013). The rhizosphere microbiome: significance of plant beneficial, plant pathogenic, and human pathogenic microorganisms. FEMS Microbiol. Rev..

[21] Vandenkoornhuyse P (2015). The importance of the microbiome of the plant holobiont. New Phytol..

[22] Lemanceau P, Blouin M, Muller D, Moenne-Loccoz Y (2017). Let the core microbiota be functional. Trends Plant Sci..

[23] Latour X (1999). The establishment of an introduced community of fluorescent pseudomonads is affected both by the soil-type and the rhizosphere. FEMS Microbiol. Ecol..

[24] Schreiter S (2014). Effect of the soil type on the microbiome in the rhizosphere of field-grown lettuce. Front. Microbiol..

[25] Colin Y (2017). Taxonomic and functional shifts in the beech rhizosphere microbiome across a natural soil toposequence. Sci. Rep..

[26] Kareiva P, Watts S, McDonald R, Boucher T (2007). Domesticated nature: shaping landscapes and ecosystems for human welfare. Science.

[27] Ellis EC (2013). Used planet: A global history. Proc. Natl. Acad. Sci. USA.

[28] Stone D (2016). A method of establishing a transect for biodiversity and ecosystem function monitoring across Europe. Appl. Soil. Ecol..

[29] Delgado‐Baquerizo M (2017). It is elemental: soil nutrient stoichiometry drives bacterial diversity. Environ. Microbiol..

[30] Delgado-Baquerizo M (2018). A global atlas of the dominant bacteria found in soil. Science.

[31] Terrat S (2017). Mapping and predictive variations of soil bacterial richness across France. PLoS One.

[32] Thompson LR (2017). A communal catalogue reveals Earth’s multiscale microbial diversity. Nature.

[33] Lienhard P (2014). Pyrosequencing evidences the impact of cropping on soil bacterial and fungal diversity in Laos tropical grassland. Agron. Sustain. Dev..

[34] Constancias F (2015). Mapping and determinism of soil microbial community distribution across an agricultural landscape. Microbiologyopen.

[35] Kuramae EE (2012). Soil characteristics more strongly influence soil bacterial communities than land‐use type. FEMS Microbiol. Ecol..

[36] Karimi, B. *et al*. Biogeography of soil bacteria and archaea across France. *Sci Adv* (in press). 10.1126/sciadv.aat1808 PMC6031370 29978046

[37] Fierer N, Bradford MA, Jackson RB (2008). Toward an ecological classification of soil bacteria. Ecology.

[38] Green JL (2004). Spatial scaling of microbial eukaryote diversity. Nature.

[39] Horner-Devine MC, Lage M, Hughes JB, Bohannan BJM (2004). A taxa-area relationship for bacteria. Nature.

[40] Barreto DP, Conrad R, Klose M, Claus P, Enrich-Prast A (2014). Distance-decay and taxa-area relationships for bacteria, archaea and methanogenic archaea in a tropical lake sediment. Plos One.

[41] Tu Q (2016). Biogeographic patterns of soil diazotrophic communities across six forests in the North America. Mol. Ecol ..

[42] Zinger L, Boetius A, Ramette A (2014). Bacterial taxa-area and distance-decay relationships in marine environments. Mol. Ecol..

[43] Terrat S (2015). Improving soil bacterial taxa–area relationships assessment using DNA meta-barcoding. Heredity.

[44] Powell JR (2015). Deterministic processes vary during community assembly for ecologically dissimilar taxa. Nat. Commun..

[45] Van Der Gast C (2014). Microbial biogeography: the end of the ubiquitous dispersal hypothesis?. Environ. Microbiol..

[46] Griffiths RI (2016). Mapping and validating predictions of bacterial biodiversity using European and national scale datasets. Appl. Soil Ecol..

[47] Hanson C, Fuhrman J, Horner-Devine MC, Martiny JBH (2012). Beyond biogeographic patterns: processes shaping the microbial landscape. Nat. Rev. Microbiol..

[48] Hermans SM (2016). Bacteria as emerging indicators of soil condition. Appl. Environ. Microbiol..

[49] Banerjee, S., Schlaeppi, K. & van der Heijden, M.G.A. Keystone taxa as drivers of microbiome structure and functioning. *Nat. Rev. Microbiol*. **16**, 10.1038/s41579-018-0024-1 (2018). 10.1038/s41579-018-0024-1 29789680

[50] Keith AM (2012). Cross-taxa congruence, indicators and environmental gradients in soils under agricultural and extensive land management. Eur. J. Soil Biol..

[51] Van Horn DJ (2013). Factors controlling soil microbial biomass and bacterial diversity and community composition in a cold desert ecosystem: role of geographic scale. PLoS One.

[52] Constancias F (2015). Contrasting spatial patterns and ecological attributes of soil bacterial and archaeal taxa across a landscape. Microbiologyopen.

[53] Bergmann GT (2011). The under-recognized dominance of *Verrucomicrobia* in soil bacterial communities. Soil. Biol. Biochem..

[54] Kuramae EE (2012). Soil characteristics more strongly influence soil bacterial communities than land-use type. FEMS Microbiol. Ecol..

[55] Acosta-Martinez V, Dowd S, Sun Y, Allen V (2008). Tag-encoded pyrosequencing analysis of bacterial diversity in a single soil type as affected by management and land use. Soil Biol. Biochem..

[56] da C Jesus E (2009). Changes in land use alter the structure of bacterial communities in Western Amazon soils. ISME J..

[57] Gardi C (2009). Soil biodiversity monitoring in Europe: ongoing activities and challenges. Eur. J Soil. Sci..

[58] Creamer RE (2016). Ecological network analysis reveals the inter-connection between soil biodiversity and ecosystem function as affected by land use across Europe. Appl. Soil Ecol..

[59] ISO (International Organization for Standardization). Soil Quality - Determination of particle size distribution in mineral soil – Method by sieving and sedimentation. ISO 11277, Geneva, Switzerland (1998).

[60] ISO (International Organization for Standardization). Soil quality - Determination of organic and total Carbon after dry combustion (elementary analysis). ISO 10694, Geneva, Switzerland (1995).

[61] Massey, P. *et al*. Irish Soil Information System: Laboratory Standard Operating Procedures. Technical Report (2007-S-CD-1-S1), published online by Environmental Protection Agency, Ireland. Preprint at https://erc.epa.ie/safer/iso19115/ displayISO19115jsp?isoID=3062 (2014).

[62] ISO (International Organization for Standardization). Soil quality - Determination of effective cation exchange capacity and base saturation level using barium chloride solution. ISO 11260, Geneva, Switzerland (1994).

[63] van Reeuwijk, L. P. Procedures for soil analysis, 6^th^ Ed. Technical Paper 9, International Soil Reference and Information Centre, FAO (2002).

[64] McDonald NT (2014). Evaluation of soil tests for predicting nitrogen mineralization in temperate grassland soils. Soil Sci. Soc. Am. J..

[65] Mehlich A (1984). Mehlich-3 soil test extractant: a modification of Mehlich- 2 extractant. Commun. Soil. Sci. Plant Anal..

[66] Plassart P (2012). Evaluation of the ISO standard 11063 DNA extraction procedure for assessing soil microbial abundance and community structure. PLoS One.

[67] Terrat S (2012). Molecular biomass and MetaTaxogenomic assessment of soil microbial communities as influenced by soil DNA extraction procedure. Microb. Biotechnol..

[68] R Core Team. R: A language and environment for statistical computing. R Foundation for Statistical Computing, Vienna, Austria. http://www.R-project.org (2013).

[69] Dray S, Dufour AB (2007). The ade4 package: implementing the duality diagram for ecologists. J. Stat. Softw..

[70] Oksanen J. *et al*. Vegan: Community ecology. Preprint at http://ftp.uni-bayreuth.de/math/statlib/R/CRAN/doc/packages/vegan.pdf (2015).

[71] De Cáceres M, Legendre P (2009). Associations between species and groups of sites: indices and statistical inference. Ecology.

[72] Harte J, Kinzig A, Green J (1999). Self-similarity in the distribution and abundance of species. Science.

[73] Lozupone C, Knight R (2005). UniFrac: a new phylogenetic method for comparing microbial communities. Appl. Environ. Microbiol..

[74] Hartigan JA, Wong MA (1979). A K-means clustering algorithm. J. R. Stat. Soc. Ser. C Appl. Stat..

