# Supplementary material for: Soil parameters, land use, and geographical distance drive soil bacterial communities along a European transect
Source: Sci Rep. 2019 Jan 24;9:605. doi: 10.1038/s41598-018-36867-2 (PMC6345909; doi:10.1038/s41598-018-36867-2)

**Supplementary Information**

**Soil parameters, land use, and geographical distance drive soil bacterial communities along a European transect**

Pierre Plassart^1*^, Nicolas Chemidlin Prévost-Bouré^1*^, Stéphane Uroz^2*^, Samuel Dequiedt^1^, Dorothy Stone^3^, Rachel Creamer^3,4^, Robert I. Griffiths^5^, Mark J. Bailey^5^, Lionel Ranjard^1^, and Philippe Lemanceau^1^

^1^Agroécologie, AgroSup Dijon, INRA, Univ. Bourgogne, Univ. Bourgogne Franche-Comté, F-21000 Dijon, France

^2^UMR 1136 Interactions Arbres Micro-organismes, INRA Univ Lorraine, F-54280 Champenoux*,* France

^3^TEAGASC, Johnstown Castle, Wexford, Ireland

^4^Present address: Wageningen University and Research, Wageningen, The Netherlands

^5^Centre for Ecology & Hydrology, Benson Lane, Crowmarsh Gifford, Wallingford, UK

*These authors contributed equally

**Supplementary data S1** Bioinformatic parameters used in the analysis of bar-coded pyrosequencing results

| Step | Parameter |  |
| --- | --- | --- |
| Preprocessing | Length threshold | 370 |
|  | Ambiguities tolerated | 0 |
|  | Detection of proximal primer sequence | Complete and perfect |
|  | Detection of distal primer sequence | No |
| Clustering | Similarity level | 95% |
|  | Differences in homopolymer length | Ignored |
| Filtering | Clustering similarity threshold | 95% |
| Homogenization | Reads per sample | 8085 |
| Analysis | Similarity level | 95% |
|  | Differences in homopolymer length | Ignored |
|  | Computation of a UNIFRAC distance matrix | Yes |

**Supplementary data S2. Relative abundances of the bacterial and archaeal phyla** within the European transect sampling. “Environmental” refers to sequences found in environmental samples already included in public databases, but not taxonomically assigned; “Unclassified” refers to sequences unassigned at the phylum level, but for which a taxonomic assignation is available for other levels (order, family, …); “Unknown” refers to sequences for which no taxonomic assignation is available.

|  | Relative abundance (%) | | |
| --- | --- | --- | --- |
|  | Minimum | Maximum | Average |
| Proteobacteria | 37.34 | 71.18 | 56.40 |
| Actinobacteria | 4.02 | 27.84 | 15.66 |
| Acidobacteria | 3.65 | 14.20 | 7.10 |
| Bacteroidetes | 0.84 | 19.08 | 6.56 |
| Firmicutes | 0.48 | 10.24 | 4.49 |
| Planctomycetes | 2.03 | 6.42 | 3.48 |
| Chloroflexi | 0.00 | 8.50 | 2.30 |
| Nitrospirae | 0.02 | 2.78 | 0.98 |
| Gemmatimonadetes | 0.00 | 3.19 | 0.78 |
| Verrucomicrobia | 0.16 | 2.07 | 0.67 |
| Chlorobi | 0.00 | 1.63 | 0.36 |
| Armatimonadetes | 0.00 | 0.49 | 0.13 |
| Elusimicrobia | 0.00 | 0.54 | 0.12 |
| Fibrobacteres | 0.00 | 0.53 | 0.11 |
| Thaumarchaeota | 0.00 | 1.58 | 0.08 |
| Crenarchaeota | 0.00 | 1.16 | 0.07 |
| Cyanobacteria | 0.00 | 0.25 | 0.03 |
| TM6 | 0.00 | 0.49 | 0.02 |
| Spirochaetes | 0.00 | 0.24 | 0.02 |
| Deinococcus-Thermus | 0.00 | 0.69 | 0.01 |
| Synergistetes | 0.00 | 0.07 | 0.01 |
| Tenericutes | 0.00 | 0.09 | 0.01 |
| Dictyoglomi | 0.00 | 0.09 | 0.01 |
| Nitrospinae | 0.00 | 0.07 | 0.01 |
| Chlamydiae | 0.00 | 0.09 | 0.01 |
| Thermodesulfobacteria | 0.00 | 0.04 | 0.00 |
| Euryarchaeota | 0.00 | 0.02 | 0.00 |
| Fusobacteria | 0.00 | 0.01 | 0.00 |
| Lentisphaerae | 0.00 | 0.01 | 0.00 |
| Deferribacteres | 0.00 | 0.01 | 0.00 |
| Aquificae | 0.00 | 0.00 | 0.00 |
| Caldiserica | 0.00 | 0.00 | 0.00 |
| Chrysiogenetes | 0.00 | 0.00 | 0.00 |
| Ignavibacteria | 0.00 | 0.00 | 0.00 |
| Korarchaeota | 0.00 | 0.00 | 0.00 |

**Supplementary Figure 1. NMDS ordination of soil bacterial communities assessed using pyrosequencing**, derived from weighted UniFrac distances. Coloured circles respectively represent site scores for soils according to their country of origin. The stress value was <0.2 which indicates that these data were well-represented by the two dimensional representation.


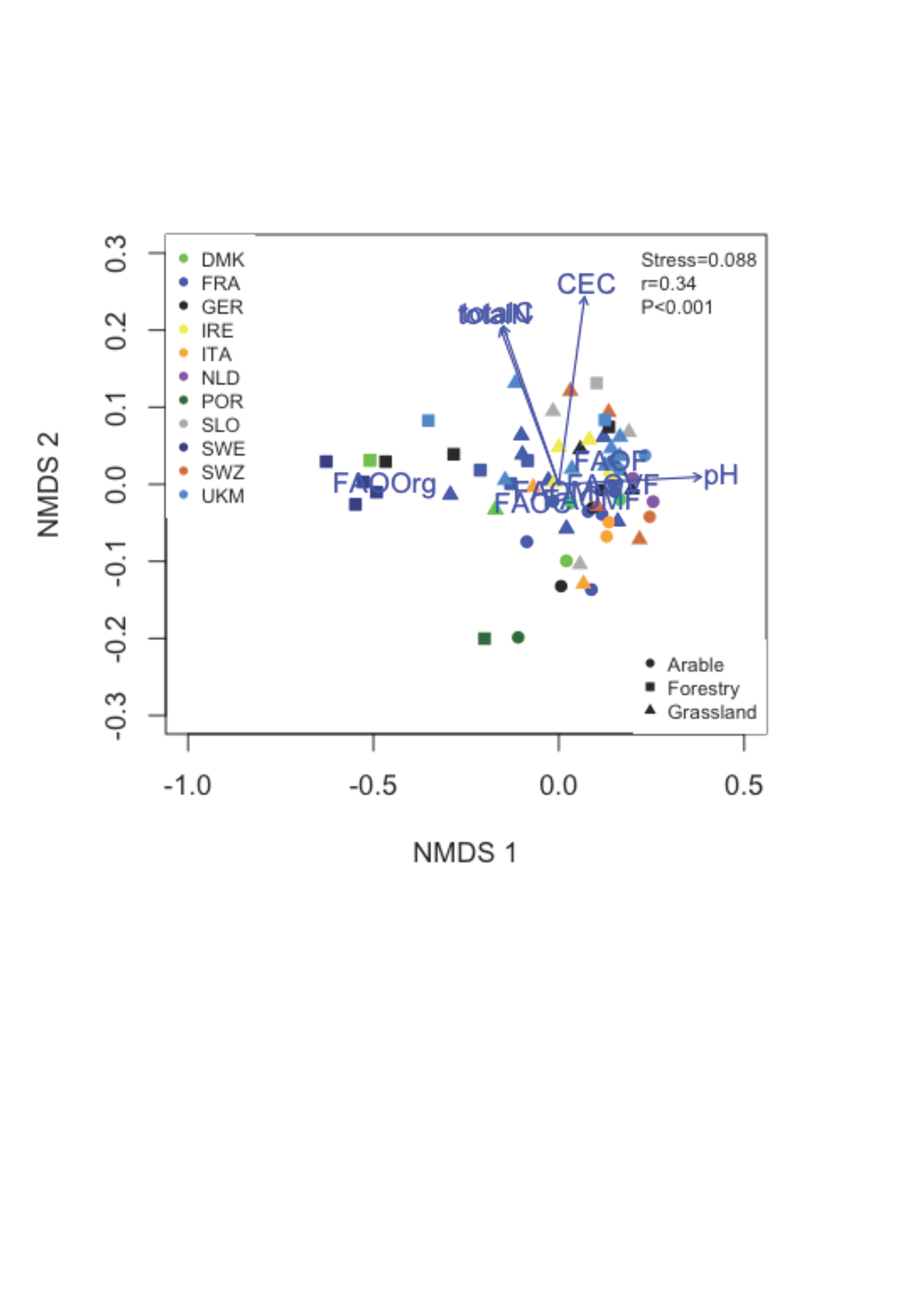

Supplement: Supplementary file 1 — Supplementary Information [file 41598_2018_36867_MOESM1_ESM.docx]
